# Genome-wide analyses of direct target genes of four rice NAC-domain transcription factors involved in drought tolerance

**DOI:** 10.1186/s12864-017-4367-1

**Published:** 2018-01-12

**Authors:** Pil Joong Chung, Harin Jung, Yang Do Choi, Ju-Kon Kim

**Affiliations:** 1 Graduate School of International Agricultural Technology and Crop Biotechnology Institute/GreenBio Science & Technology, Seoul National University, Pyeongchang, 25354 South Korea; 2 Department of Agricultural Biotechnology, Seoul National University, Seoul, 08826 South Korea; 3 Present address: NUS Synthetic Biology for Clinical and Technological Innovation (SynCTI), Department of Biochemistry, Yong Loo Lin School of Medicine, National University of Singapore, Singapore, 117596 Singapore

**Keywords:** Drought tolerance, Genome-wide analysis, NAC transcription factors, *Oryza sativa*, RNA-Seq, ChIP-Seq

## Abstract

**Background:**

Plant stress responses and mechanisms determining tolerance are controlled by diverse sets of genes. Transcription factors (TFs) have been implicated in conferring drought tolerance under drought stress conditions, and the identification of their target genes can elucidate molecular regulatory networks that orchestrate tolerance mechanisms.

**Results:**

We generated transgenic rice plants overexpressing the 4 rice TFs, *OsNAC5, 6, 9,* and *10*, under the control of the root-specific *RCc3* promoter. We showed that they were tolerant to drought stress with reduced loss of grain yield under drought conditions compared with wild type plants. To understand the molecular mechanisms underlying this tolerance, we here performed chromatin immunoprecipitation (ChIP)-Seq and RNA-Seq analyses to identify the direct target genes of the OsNAC proteins using the *RCc3:6MYC-OsNAC* expressing roots. A total of 475 binding loci for the 4 OsNAC proteins were identified by cross-referencing their binding to promoter regions and the expression levels of the corresponding genes. The binding loci were distributed among the promoter regions of 391 target genes that were directly up-regulated by one of the OsNAC proteins in four *RCc3:6MYC-OsNAC* transgenic lines. Based on gene ontology (GO) analysis, the direct target genes were related to transmembrane/transporter activity, vesicle, plant hormones, carbohydrate metabolism, and TFs. The direct targets of each OsNAC range from 4.0–8.7% of the total number of up-regulated genes found in the RNA-Seq data sets. Thus, each OsNAC up-regulates a set of direct target genes that alter root system architecture in the *RCc3:OsNAC* plants to confer drought tolerance. Our results provide a valuable resource for functional dissection of the molecular mechanisms of drought tolerance.

**Conclusions:**

Many of the target genes, including transmembrane/transporter, vesicle related, auxin/hormone related, carbohydrate metabolic processes, and transcription factor genes, that are up-regulated by OsNACs act as the cellular components which would alter the root architectures of *RCc3:OsNAC*s for drought tolerance.

**Electronic supplementary material:**

The online version of this article (10.1186/s12864-017-4367-1) contains supplementary material, which is available to authorized users.

## Background

The global population is approximately 7.2 billion, but is projected to reach 9.7 billion by 2050 [1]. In order to feed this growing population, it is estimated that current the annual crop production of 2 billion tons will need to double within this time frame. Plants are constantly exposed to environmental stresses, such as drought, salt, flooding, and high temperatures, which cause severe losses of crop yield. Drought stress, in particular, adversely affects plant growth, membrane integrity, osmotic adjustments, water relations, photosynthetic activity and grain yield [2]. New innovative approaches are need to develop genetically engineered crops that are tolerant to drought stress conditions. Accordingly, an understanding of the molecular and cellular responses to drought stress is a prerequisite for the identification of key regulators that can be used to produce such crops [3–5].

Molecular and genomic analyses have revealed many drought-inducible transcription factors (TFs) that regulate the expression of stress-inducible downstream genes [6, 7]. These include TFs belonging to the AP2/ERF, MYB, bZIP and NAC families. TFs are regulators that bind to *cis*-regulatory sequences in the promoter and/or enhancer elements of target genes to activate or inactivate their expression [8]. NAC [no apical meristem (NAM), *Arabidopsis thaliana* transcription activation factor (ATAF1/2) and cup shaped cotyledon (CUC2)] domain containing genes constitutes one of the largest plant-specific TF families in both *A. thaliana* and rice [9, 10]. NAC TFs have been shown to play a role in diverse processes involving shoot apical meristem development, embryogenesis, lateral root development [11–13], plant defense mechanisms, senescence [14], and abiotic stress responses [15]. It is estimated that 2408 genes encode TF or TF-like proteins in rice (*Oryza sativa*), based on the Plant Transcription Factor Database v3.0 (http://planttfdb.cbi.pku.edu.cn). Among these, rice has 170 putative OsNAC genes, based on sequence analysis (Plant Transcription Factor Database, http://planttfdb.cbi.pku.edu.cn/), of which 41 and 29 are induced and repressed, respectively, in leaves of reproductive stage under drought stress conditions [16]. However, only a few of these genes have been functionally characterized in the context of drought tolerance, although it is known that they are often functionally redundant [17, 18]. Despite their important function in modulating drought tolerance, little is known about the molecular mechanisms associated with OsNAC TFs and their target genes [19].

In *A. thaliana*, some of the NAC TF direct targets have been identified [20]. Ohashi-Ito et al. (2010) reported that VND6 (VASCULAR-RELATED NAC-DOMAIN6) is a direct regulator of *XCP1 (XYLEM CYSTEINE PROTEASE1),* and that IRX5/*CESA4 (IRREGULARXYLEM5/CELLULOSE SYNTHASE)* is involved in plant cell death and secondary wall formation [21]. In addition, the *A. thaliana* NAC TF, VNI2 (VND INTERACTING2) regulates leaf longevity under environmental stress conditions through direct binding to the promoters of the *COR* (*COLD-REGULATED*) and *RD* (*RESPONSIVE TO DEHYDRATION*) genes [22].

Chromatin immunoprecipitation (ChIP) can be used as a genome-wide approach to verify in vivo interactions between TF proteins and genomic DNA under physiological conditions, and to estimate the density of TF binding at specific loci [23]. Recently, both RNA-Seq and ChIP-Seq technologies have been widely used to measure the transcript levels of TFs and to obtain information about genome-wide binding sites, respectively [24]. Such approaches can provide insights into changes in protein association with regulatory regions of genes in response to environmental clues, and can help connect those changes with the transcription patterns of target genes.

Previously, we demonstrated that root-specific overexpression of each of four TF genes, OsNAC5, 6, 9, and 10, significantly enhanced drought tolerance, leading to high grain yield under field-drought conditions [25, 29–31]. The common phenotypic characteristics of each OsNAC overexpressing transgenic rice showed a change in root architecture. In particular, enlarged stele and aerenchyma (OsNAC5, OsNAC6 and OsNAC9), and enlarged stele, cortex, and epidermis (OsNAC10) can explain the altered root architecture, including root number and root diameter, and ultimately conferring increased drought tolerance phenotype. In this study, we used genome-wide ChIP-Seq and RNA-Seq analyses to identify the direct target genes for four OsNAC TFs that have been reported to be involved in influencing root growth, leading to drought tolerance. Our results reveal the regulatory networks governed by OsNAC TFs that contribute to drought-tolerance mechanisms, providing a foundation for the development of drought tolerant crops.

## Methods

### Plasmid construction and rice transformation

To generate *RCc3:6MYC-OsNAC* rice lines, the coding sequence of OsNAC genes were amplified using PrimeSTAR DNA polymerase (TaKaRa, Tokyo, Japan) with the respective gene-specific forward and reverse primers. After enzyme digestion of the PCR products with *Bam*HI and *Not*I, the DNA fragment containing the coding sequence was then ligated into the multiple cloning site of the pE3n vector, which is flanked with a 6MYC tag coding sequence [32]. Finally, the 6MYC-OsNAC sequences from the pE3n-OsNAC was cloned into the p700-RCc3 vector carrying a 1.3 kb RCc3 (Os02g0662000) promoter sequence using the Gateway system (Invitrogen, Carlsbad, CA) [29], and the final vectors were named *RCc3:6MYC-OsNAC*. Transgenic plants were obtained by *Agrobacterium tumefaciens* (strain LBA4404)-mediated transformation of embryogenic rice callus (*Oryza sativa L. japonica* cv. Illmi), as previously described [33].

### Plant growth and sampling

*RCc3:6MYC-OsNAC* transgenic and non-transgenic (NT; *Oryza sativa L. japonica* cv. Illmi) seeds were germinated on MS (Murashige and Skoog) medium in the dark at 28 °C for 3 d (days) and transferred into light conditions for a half day. Seedlings were transplanted into the soil pots (4 × 4 × 6 cm; 3 plants per pot) and grown in a greenhouse (30 °C for 16 h for day/ 25 °C for 8 h for night cycle) under 60–80% humidity and 200-500 umol m^-2^ s^-1^ light intensity (37°32΄51.3”N 128°26΄26.6″E). Two weeks after transplanting to soil, the seedlings were completely submerged in water in the greenhouse for 3 d, the roots were harvested, immediately frozen in liquid nitrogen and stored at −80 °C. The root tissues for RNA-Seq and ChIP-Seq were simultaneously prepared as independent biological replicates.

### Confirmation of expression of the OsNAC fused 6MYC protein using anti-MYC antibody

Proteins were extracted from the root samples with extraction buffer [50 mM Tris·HCl (pH 8.0), 150 mM NaCl, 5 mM EDTA, and 0.2% Triton X-100], and protein concentrations were determined using the Bradford method (Bio-Rad Laboratories, Inc., http://www.bio-rad.com). Protein extracts were then separated on 10% SDS-polyacrylamide gels and blotted onto a polyvinylidene difluoride (PVDF) membrane (Immobilon-P; Millipore Corporation, http://www.millipore.com). The immunoreactive proteins were detected using primary antibodies against c-MYC (#sc-789; Santa Cruz Biotechnology), and detected by chemiluminescence with Pierce Super Signal Substrate (Pierce, http://www.piercenet.com) in accordance with the manufacturer’s protocol.

### RNA-Seq

Total RNA was prepared from the root tissue of the *RCc3:6MYC-OsNAC* transgenic and NT rice plants using Trizol reagent (Invitrogen, Carlsbad, CA) and then purified with an RNeasy Mini Kit (Qiagen, Valencia, CA). Contaminating genomic DNA was removed by treating with DNase I (Invitrogen), according to the manufacturer’s instructions. Handling of the RNA-Seq library construction, next-generation sequencing (NGS) and DEG (Differential Expressed Gene) analysis was completed by MACROGEN Inc., Seoul, Korea (http://macrogen.com). Raw sequence reads were trimmed to remove adaptor sequences, contaminant DNA, and PCR duplicates, and those with a quality lower than Q20 were removed. All reads were assembled with TopHat software, using annotated genes from the rapdb database [http://rapdb.dna.affrc.go.jp; IRGSP (v 1.0)]. DEGs were defined by an expression change ≥2-fold with a *P* value <0.05. The entire sets of original RNA-Seq data have been deposited in the NCBI Gene Expression Omnibus under GEO Series number GSE102919.

### Quantitative real-time PCR qRT-PCR

To validate the results of the RNA-Seq analysis, total 23 genes, representing up-regulated, unchanged, and down-regulated, were selected from the DEGs (differentially expressed genes) in the RNA-Seq analysis (*P* value < 0.05). RNA isolated from the roots of NT, OsNAC5, 6, 9, and 10 transgenic rice lines was used for cDNA synthesis and qRT-PCR, using gene specific primers. The primers were designed to amplify 120–200 bp amplicons with a T_m_ at 60 °C. First-strand cDNA was synthesized from 1.0 μg of total RNA primed with oligo-dT using Superscript III (Invitrogen). qRT-PCR was performed using a Stratagene Mx3000P DNA analyzer (Agilent Technologies) with the Solg™ 2x real-time PCR smart mix with evagreen (Solgent, Seoul, Korea) for signal detection. To normalize the total amount of cDNA present in each reaction, a housekeeping gene encoding ubiquitin (Os06g0681400) was co-amplified. Primer sequences are listed in Additional file 1: Table S8. Values are the means ± SD (standard deviation) of three independnet experiments.

### Chromatin immunoprecipitation (ChIP) and ChIP-Seq

Nuclei-enriched fractions were partially purified from rice roots as previously described with slight modifications [26]. *RCc3:OsNACs* and NT rice seedlings grown in soil were hydroponically adapted in water for 3 d and cross-linked with 1% formaldehyde by vacuum infiltration for 15 min. Cross-linking was stopped by the addition of a final concentration of 125 mM glycine and vacuum infiltrating for 10 min. After washing the plants in cold water, the roots were excised, frozen in liquid nitrogen and stored at −80 °C.

Chromatin from the nuclei-enriched fractions was fragmented to an average size of approximately 100–200 bp by 15 cycles of sonication (30 s each) in 15 mL falcon tubes using a Bioruptor UCD-200 sonicator (Diagenode, USA). To determine the concentration of sheared genomic DNA, an aliquot (50 μL) of chromatin solution with 200 μL of elution buffer (1% SDS and 0.1 mM NaHCO_3_) was used to reverse the cross-linking of the chromatin fractions, and the eluent was mixed with NaCl to a final concentration of 0.3 M, then incubated at 65 °C for 6 h. After reversal of the cross-links, the resulting sheared DNA was purified using the QIAquick PCR purification kit (Qiagen 28,106) and was quantified using NANO drop.

For the ChIP assays with chromatin enriched fraction, containing 5 μg of sheared DNA, immunoprecipitation was performed overnight with polyclonal anti-MYC (sc-789, Santa Cruz). Protein A agarose beads bearing immunoprecipitates were then subjected to sequential washes, and DNA was purified using the QIAquick PCR purification kit (Qiagen 28,106). Purified DNA was used to prepare libraries with the TruSeq ChIP Library preparation Kit (Illumina, Inc), and purified using the Agencourt AMPure XP system (Beckman Coulter, Inc). Sequencing was performed on an Illumina Hi-Seq 2500 with 101 bp single end reads (MACROGEN, Seoul). The ChIP-Seq data have been deposited in the NCBI Gene Expression Omnibus under GEO Series number GSE102920.

### Protoplast isolation and transactivation assay

Rice protoplasts were isolated according to the method described for *A. thaliana* by Yoo et al. (2007) and the method described for rice by Chen et al. (2006) and Zhang et al. (2011) with some modifications [27, 28, 34]. Rice seeds were grown on MS medium at 28 °C for 10 days, then leaves and stems were cut into 0.5 mm pieces using razors and plasmolyzed in a 0.6 M mannitol solution for 20 min. The plasmolyzed tissue was transferred to enzyme solution [1.5% Cellulase R-10 (Yakult Pharmaceutical Ind. Co., Japan), 0.75% Macerozyme R-10 (Yakult Pharmaceutical Ind. Co., Japan), 0.6 M mannitol, 0.1% bovine serum albumin (BSA), 1 mM CaCl_2_, 5 mM β-mercaptoethanol and 10 mM MES (pH 5.7)]. After incubation at 25 °C for 4 h with gentle shaking in the dark, an equal volume of W5 solution [154 mM NaCl, 125 mM CaCl_2_, 5 mM KCl, 5 mM D-glucose and 2 mM MES (pH 5.7)] was added and the protoplasts were collected by centrifugation at 300×*g* for 8 min. Protoplast-enriched pellets were resuspended in MMG solution [0.6 M mannitol, 15 mM MgCl_2_ and 4 mM MES (pH 5.7)], and 15 μl DNA (including 3 μg effector (OsNAC TFs) and 1.5 μg reporter (promoter:*fLuc* and internal control *35S:rLuc*) was used to transfect the protoplast solution harboring up to 2–3 × 10^6^ cells. Dual luciferase activity was analyzed using a Dual-Luciferase Reporter Assay System (Promega, USA) and measured with an Infinite M200 System (Tecan, USA). Three independent transfections of each sample were performed, and the relative luciferase activity was calculated as the ratio between fLuc and rLuc.

## Results

### Root-specific overexpression of four *6MYC*-tagged *OsNAC* genes

**Fig. 1:**
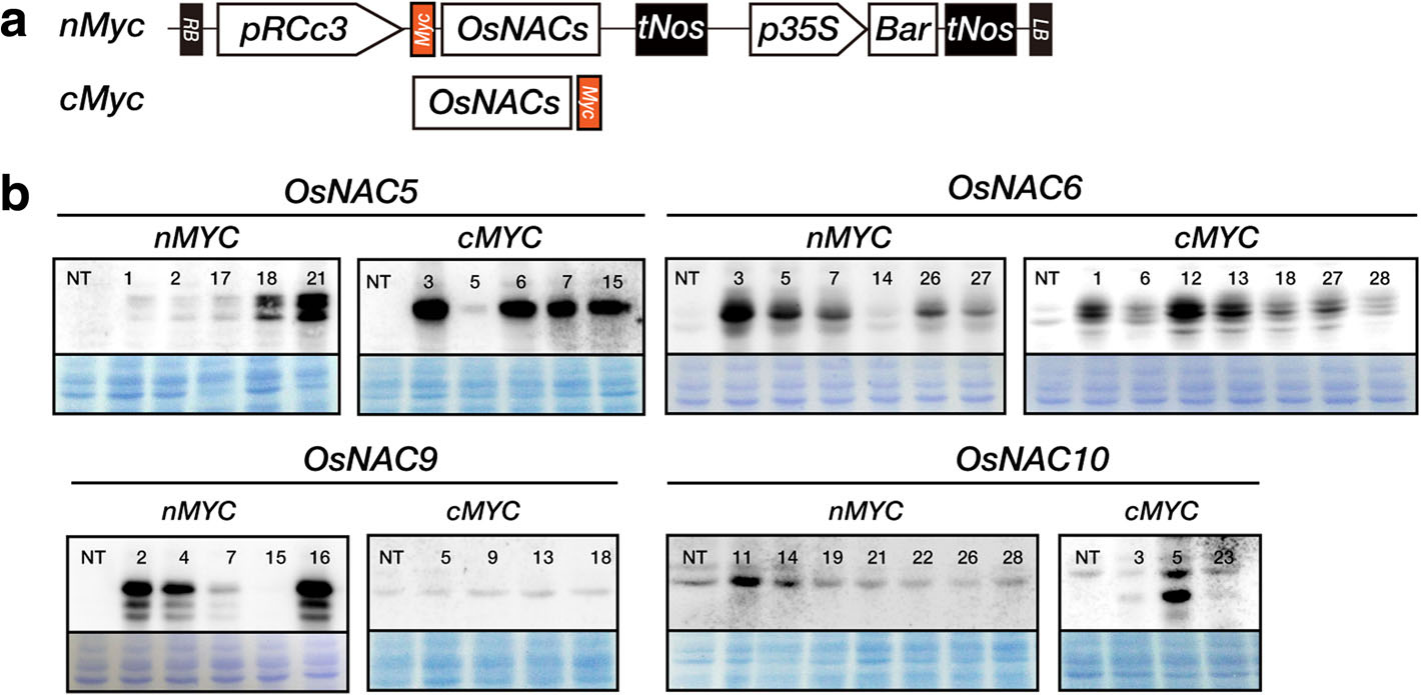
Construction of *RCc3:nMYC:OsNAC* or *RCc3:OsNAC:cMYC* transgenic rice plants. **a** Map of the binary vector used for generating *RCc3:OsNAC5, 6, 9,* and *10* transgenic rice lines. RB, right border; *pRCc3*, a root specific promoter [48]; *tNOS*, *NOS* terminator; *Bar*, *phosphinothricin acetyltransferase* gene, an herbicide resistance gene used as a selection marker; LB, left border. Orange color indicates nMYC (N-terminal portion of 6MYC protein) or cMYC (C-terminal portion of 6MYC protein). **b** Translational expression of OsNAC5, 6, 9, and 10 proteins fused cMYC or nMYC, as measured by Western blot analysis using an anti-MYC antibody. Protein extracts from roots of twenty-day-old nontransgenic wild type (NT, *Oryza sativa L. japonica* cv. Illmi) and *RCc3: OsNAC5, 6, 9,* and *10* transgenic seedlings (10 μg per lane) were subjected to SDS-PAGE (polyacrylamide gel electrophoresis) and Western blot analysis. Coomassie blue staining is shown to confirm equal loading. Red figure, definite overexpressed 6MYC fused OsNAC protein

To perform functional analysis of these OsNAC TFs, we generated four root-specific 6*MYC*-tagged *OsNAC* overexpressing plants (*RCc3:6MYC-OsNAC5, 6, 9* and *10*) with the coding sequences of *OsNAC5* (Os11g0184900), *OsNAC6* (Os01g0884300), *OsNAC9* (Os03g0815100), and *OsNAC10* (Os11g0126900) fused to the *6MYC* tag [32]. Five independent lines per construct were propagated to obtain T_3_ seeds. Proteins extracts from the roots of the *RCc3:6MYC-OsNAC* transgenic lines were subjected to Western blot analysis using an α-MYC antibody (Fig. 1). Transgenic lines with the highest levels of 6MYC-tagged OsNAC protein were chosen for further experiments.

### Identification of genes differentially regulated in *RCc3: 6MYC-OsNAC* roots using RNA-Seq

**Fig. 2:**
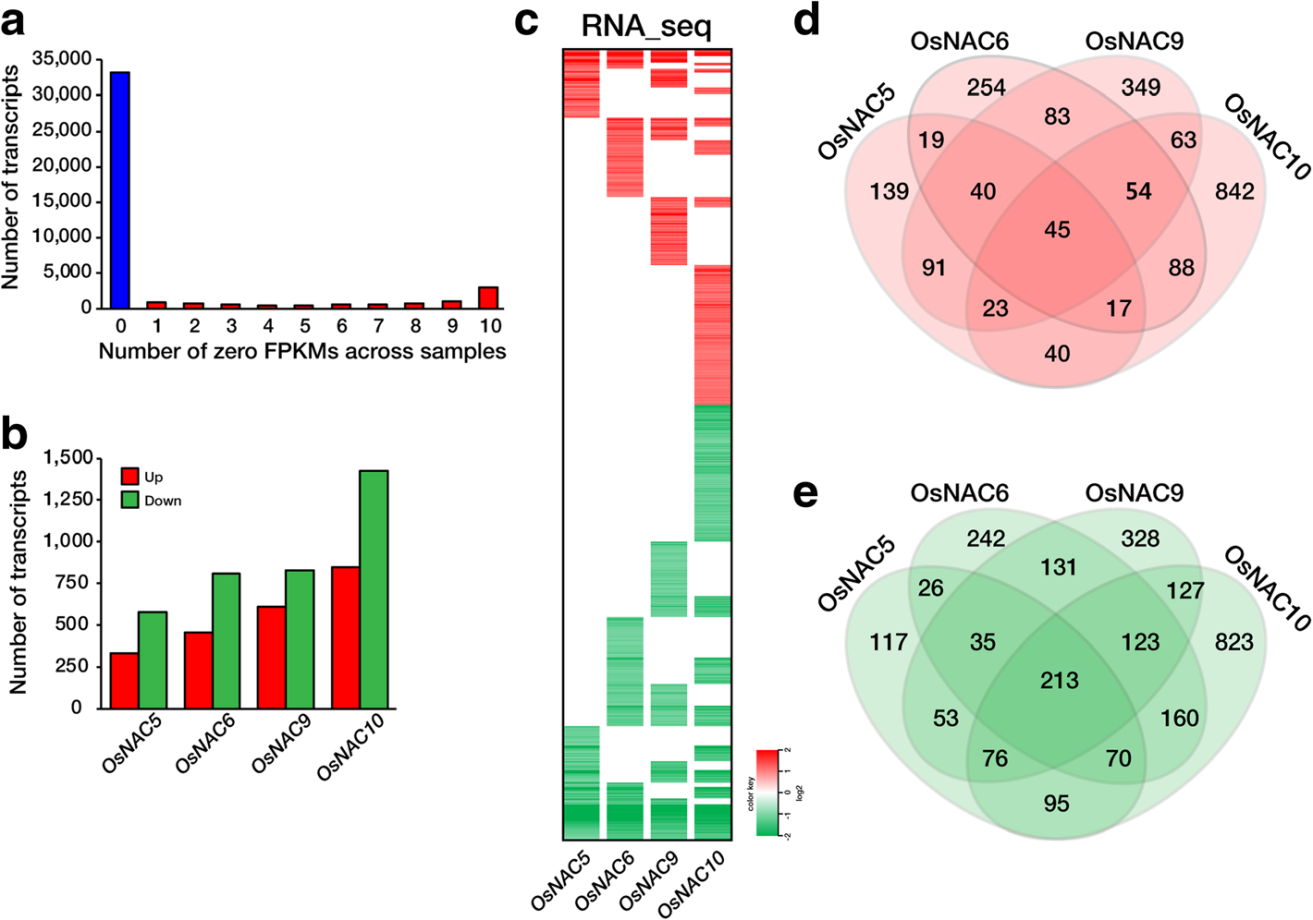
RNA-Seq data obtained by Illumina Hi-Seq 2500 sequencing. **a** Distribution of transcripts with zero fragments per kilobase of transcript per million mapped reads (FPKMs). A total of 33,178 transcripts were analyzed, following exclusion of 8457 transcripts with one or more zero FPKMs. **b** The number of statistically significant differentially expressed genes (DEGs), identified by Cufflink analysis, in various *RCc3:OsNAC* transgenic plants relative to the non-transgenic (NT) control. **c** Heat map displaying transcripts that are differentially expressed between NT and *RCc3:OsNAC5*, *6*, *9*, or *10* transgenic rice plants. Red and green color represents up-regulated and down-regulated genes, respectively. **d, e** Venn diagrams illustrating the overlapping DEGs among OsNAC5, 6, 9, and 10 transgenic plants. Numbers of DEGs that are up-regulated (**d**) and down-regulated (**e**)

**Fig. 3:**
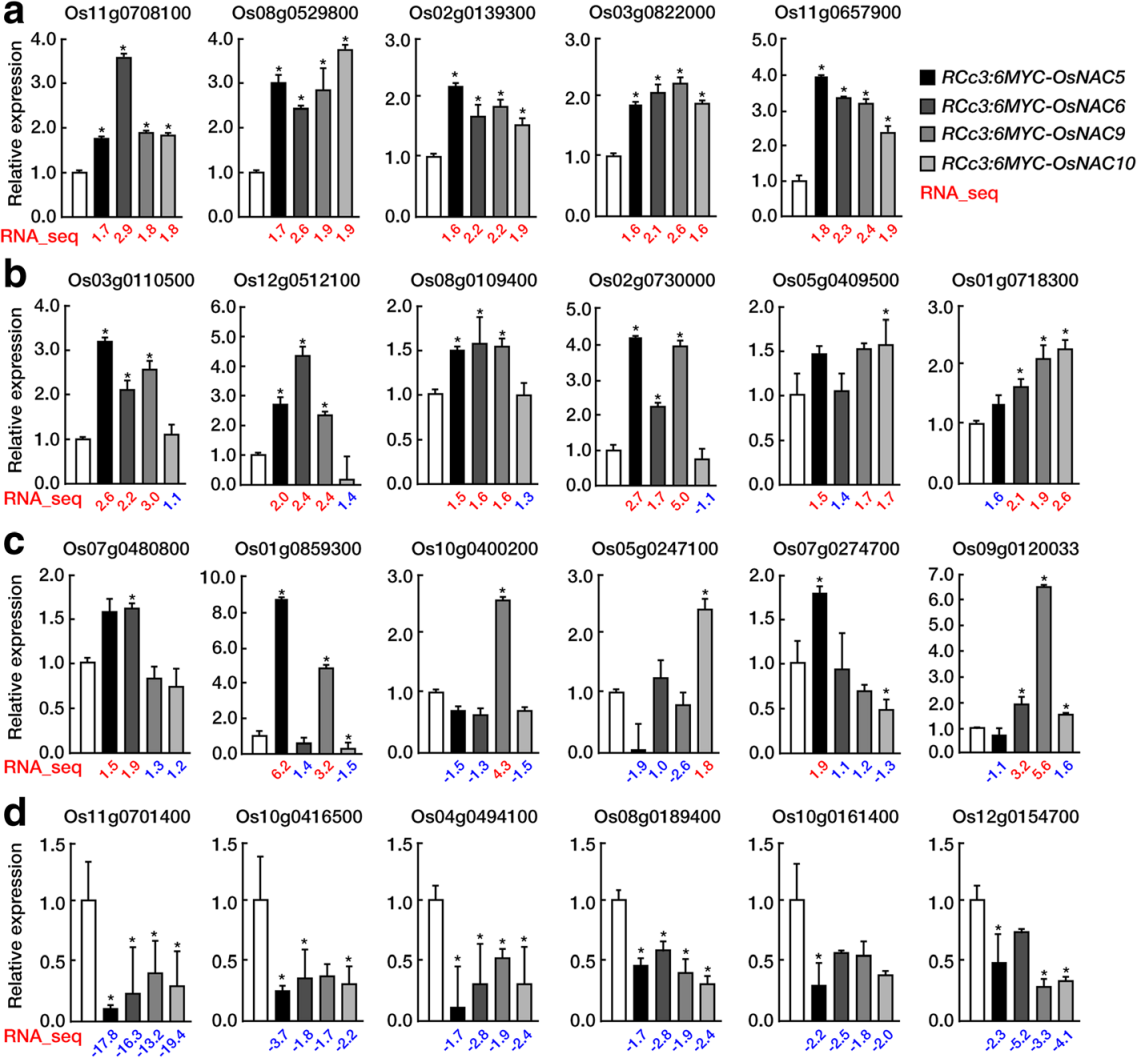
Validation of RNA-Seq expression profiles by qRT-PCR. RNA-Seq expression profiles of 24 genes between NT and *RCc3:OsNACs* transgenic rice plants were confirmed by qRT-PCR. *OsUbi1* was used as an internal control. Numbers in red (up-regulated) or blue (down-regulated) represent RNA-Seq data, whereas bars represent results of quantitative real-time PCR (qRT-PCR). Error bars indicate standard deviation. **a** Up-regulated genes in the *OsNAC5*, *6*, *9*, or *10* transgenic rice plants. **b** Up-regulated genes in 3 of the 4 *RCc3:OsNAC* transgenic rice plants. **c** Up-regulated genes in 1 or 2 of the *RCc3:OsNAC* transgenic rice plants. **d** Down-regulated genes in 4 *RCc3:OsNAC* transgenic rice lines. Data represent mean ± standard deviation (*n* = 3) and significant different from NT and OsNAC transgenic plants are indicated by asterisks (Student’s t-test, P ≤ 0.05)

To investigate OsNAC regulatory networks, RNA-Seq analyses were performed of roots from 20 d-old non-transformed (NT) and *RCc3:6MYC-OsNAC* expressing plants grown under normal growth conditions. Each data set was obtained from two biological replications. A total of 107 million raw reads were generated to identify the genes with different expression profiles, and subsequently trimmed to 102 million reads. Approximately 45 million reads were aligned to the reference rice genome (IRGSP v.1.0, http://rapdb.dna.affrc.go.jp) and 88.4%–91.6% of the reads were well matched (Additional file 2: Table S1). After read mapping, gene expression levels were calculated as FPKM (Fragments per kilobase of transcript per million mapped reads). Of a total of 41,635 transcripts, 8457 were excluded because of a zero FPKM value in one of the four *RCc3:6MYC-OsNAC* plants, leaving 33,178 transcripts for further analysis (Fig. 2a). From the 10 independent libraries from duplicate samples of *RCc3:6MYC-OsNAC5, 6, 9, 10* expressing and NT control roots, a total of 4766 DEGs were identified using the following criteria |fc (fold change) | ≥ 1.5 and *p* value <0.05 (Fig. 2b). We identified 2147 and 2619 genes that were up- and down-regulated in one or more of *RCc3:6MYC-OsNAC* sample, respectively (Fig. 2c). Specifically, 414, 601, 748, and 1172 genes were up-regulated by more than 1.5-fold in *RCc3:6MYC-OsNAC5, 6, 9* and *10* transgenic roots, respectively, and 685, 1000, 1086, and 1687 genes were down-regulated in *RCc3:6MYC-OsNAC5, 6, 9* and *10* transgenic roots, respectively. Venn diagrams representing the distribution of single gene shared amongst the four *RCc3:6MYC-OsNAC* transgenic roots are shown in Fig. 2. Of these genes, 45 and 213 genes were up- and down-regulated, respectively, in all of the *RCc3:6MYC-OsNAC* transgenic roots (Fig. 2d and e, Additional file 3: Table S2). We validated the expression patterns of 23 genes that were up-regulated in all four (Fig. 3a), three (Fig. 3b), and one or two of the *RCc3:6MYC-OsNAC* expressing root samples (Fig. 3c), and that were down-regulated in all four *RCc3:6MYC-OsNAC* root samples (Fig. 3d**),** by qRT-PCR. These results correlated well with those of the RNA-Seq analysis (Fig. 3).

To gain insight into the possible functions of the target genes of the OsNAC TFs, Gene Ontology (GO) term enrichment analysis was performed using the web-based tool, AgriGO (Additional file 4: Table S3). The ‘membrane-bounded vesicle’, ‘apoplast’, ‘cell wall’, ‘external encapsulating structure’, and ‘extracellular region’ terms of the ‘cellular component’ category were significantly enriched for the genes up-regulated in the *RCc3:6MYC-OsNAC* roots (Additional file 4: Table S3 and Additional file 5: Table S4), suggesting an association between these subcellular areas/structures and the mechanisms of drought tolerance in the OsNAC overexpressing plants.

### Identification of OsNAC5, 6, 9, and 10 binding loci by ChIP-Seq

**Fig. 4:**
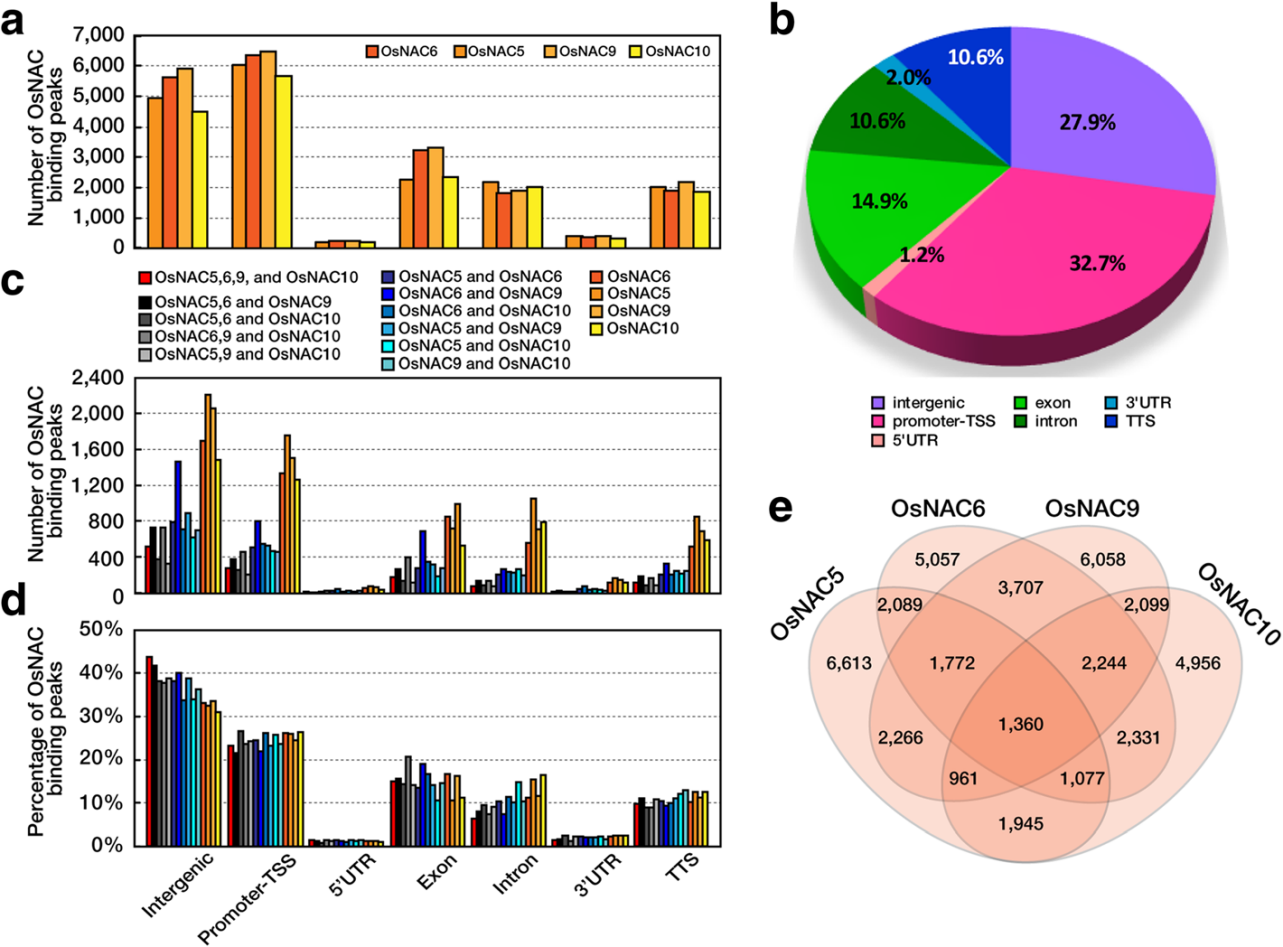
Analysis of ChIP-Seq data of OsNACs binding genomic loci distributions. Bar graph and pie charts of genomic loci distribution. **a** Bar graph of the genomic binding loci distribution of the OsNAC5, 6, 9 and 10 transcription factors. All uploaded files are combined into the same bar graph. The graph shows the number of loci distributed among intergenic, promoter, 5’UTR (untranslated region), exon, intron, 3’UTR, and TTS regions. **b** Pie chart of the genomic binding loci distribution of OsNAC5, 6, 9 and 10. This shows the percentage for each genomic location including intergenic, promoter, 5’UTR, exon, intron, 3’UTR, and TTS regions. **c, d** Distribution of OsNAC binding loci across the rice genome. Distribution of loci was plotted for each OsNAC by loci number (**c**) and by the percentage of total loci number (**d**). **e** Venn diagrams of loci shared by four OsNAC transcription factors

The RNA-Seq analyses using the four *RCc3:6MYC-OsNAC* transgenic root samples indicated both direct and indirect targets of the OsNAC proteins. To pinpoint the direct targets and to further understand how the OsNAC TFs regulate gene expression related to drought tolerance, we performed a ChIP sequencing (ChIP-Seq) analysis using DNA immunoprecipitated from roots of the four *RCc3:6MYC-OsNAC* transgenic and NT control plants. A MYC-specific antibody was used together with OsNAC bound genomic DNA fragments and ChIP DNA libraries were constructed to determine OsNAC binding sites by deep sequencing. The sequencing depth of the immunoprecipitated DNA fragments was >1 million mapped reads. Background ChIP-Seq peaks from the *RCc3:6MYC-OsNAC* roots were filtered by comparisons with those of NT control roots. The total number of read bases were calculated and are listed in Additional file 6: Table S5. Data sets were normalized to RPM (read per million) values. A total of 44,550 genomic regions were identified and annotated by comparison with the reference rice genome (IRGSP a.1.0). Figure 4a shows the distribution of genomic loci enriched by individual OsNAC proteins, such as intergenic (beyond the 10 kb region upstream of TSS, transcription start site), promoter (2 kb upstream of TSS), 5’UTR (untranslated region), exon, intron, 3’UTR, and TTS (transcription terminate site) with the numbers of enriched peaks. Interestingly, we found similar distribution patterns of genomic loci enriched by each of the four of OsNAC proteins (Fig. 4a). Overall, 72% of the binding loci were within the genic regions, including the promoter, and 28% were in intergenic regions. (Fig. 4b). Most of the binding loci were shared by four, three, and two of the OsNAC TFs (Fig. 4c and d), 1199 of which were shared by all four, and were distributed among the intergenic and promoter regions (Fig. 4d).

#### Identification of direct OsNAC targets by cross-referencing ChIP-Seq and RNA-Seq data

**Fig. 5:**
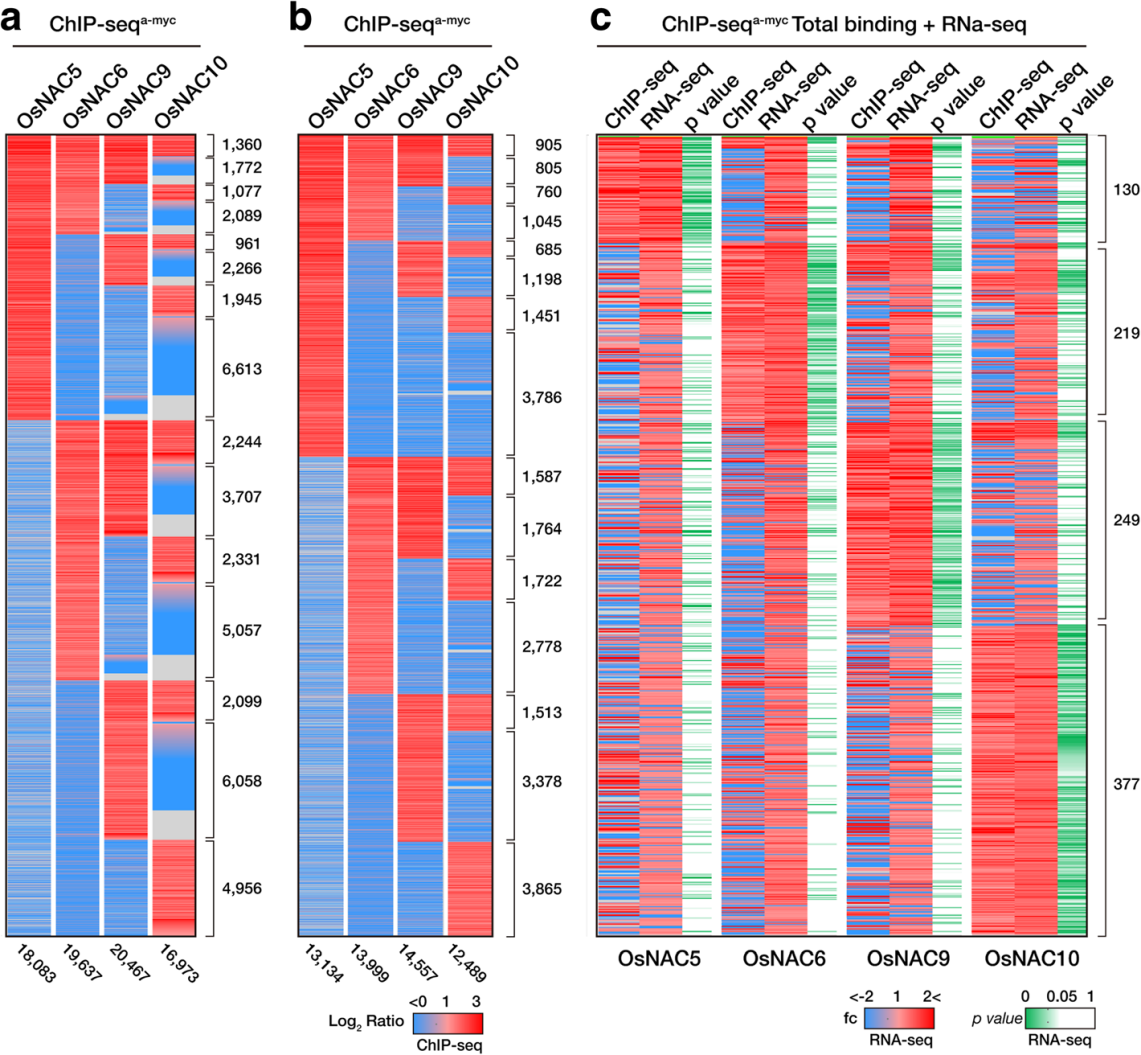
Merging of chromatin immunoprecipitation (ChIP)-Seq and RNA-Seq data sets identified direct targets of OsNAC regulatory networks. **a** The genomic distribution of OsNAC5, 6, 9 and 10 binding loci, including intergenic, promoter, 5’UTR, exon, intron, 3’UTR, and TTS regions. **b** The genomic binding loci distribution of OsNAC5, 6, 9 and 10 except intergenic regions. **c** Selected genomic loci enriched by individual OsNAC proteins with merging ChIP-Seq and RNA-Seq data sets. The Red color represents OsNAC TFs binding loci in ChIP-Seq; The Red color represents upregulated genes and blue represents downregulated genes in RNA-Seq. Green color represent *P* value in RNA-Seq

**Fig. 6:**
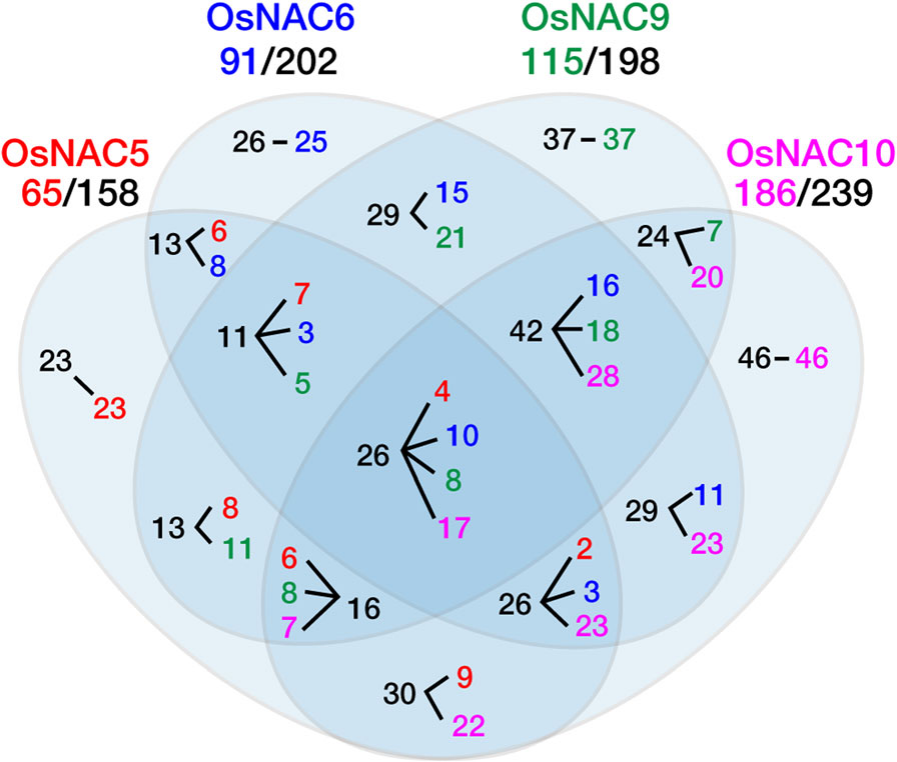
Venn diagram showing the number of direct target genes that are up-regulated either in all, or specifically in one, of the *RCc3:OsNAC5*, *6*, *9*, and *10* transgenic rice lines. Total number of promoter binding loci (black Arabic numerals) are 158, 202, 198, and 239 for OsNAC5, 6, 9, and 10, respectively. Numbers in red, blue, green, and purple indicate genes that are up-regulated in *RCc3:OsNAC5*, *6*, *9*, or *10* transgenic rice lines, respectively

We identified 18,083, 19,637, 20,467, and 16,973 enriched regions for OsNAC5, 6, 9, and 10, respectively, using ChIP-Seq (Figs. 4e and 5a). To identify the direct OsNAC target genes, we combined the ChIP-Seq and RNA-Seq data. Initially, we removed the intergenic regions from the ChIP enriched regions and obtained 13,134, 13,994, 14,557, and 12,489 enriched regions for OsNAC5, 6, 9, and 10, respectively (Fig. 5b). These were sorted for genomic loci enriched by >2.0-fold (log2 ≥ 1.0) compared with the NT control. We then combined the RNA-Seq data from individual *RCc3:6MYC-OsNAC* transgenic roots, considering genes whose expression was up-regulated by >1.5-fold (*p* value <0.05) compared with the NT roots, with the selected genomic loci enriched by individual OsNAC proteins (Fig. 5c). A total of 975 genomic loci, distributed among 460 promoters, 11 5’ UTRs, 211 exons, 116 introns, 148 TTSs, and 28 3’ UTRs, were enriched at least one of the OsNAC samples (Fig. 5c; Additional file 7: Table S6). We focused on 471 binding loci (460 promoters and 11 5’ UTRs) involving 391 genes after redundant binding loci were removed (Fig. 6).

To elucidate the functions of these 391 direct OsNAC target genes, we performed a Gene Ontology (GO) based functional enrichment analysis using AgriGO (Go analysis toolkit and database for agriculture community, http://bioinfo.cau.edu.cn/agriGO/index.php) (Additional file 8: Fig. S1). Singular enrichment analysis (SEA) was used to GO enrichment analysis by comparing the list of differentially expressed genes to all expressed genes. The ‘membrane and vesicle related cellular component including vesicle’ (GO:0031982), membrane-bound vesicle (GO: 0031988), ‘cytoplasmic vesicle’ (GO:0031410), and ‘cytoplasmic membrane-bounded vesicle’ (GO:001623) terms in the ‘cellular component’ category were significantly enriched. Additional file 9: Table S7 shows the 391 target genes affiliated with 8 GO terms in ‘cellular components’. More specifically, the candidate genes were enriched in the ‘membrane and vesicle related cellular component’ category, including 192 genes in the ‘organelle’ related GO terms, 87 genes in the ‘vesicle’ related GO terms, and 224 genes in the ‘cell related’ GO terms. Some of these were classified into the ‘transmembrane/transporter activity containing proteins’, ‘vesicle related proteins’, ‘auxin/hormone related proteins’, ‘carbohydrate metabolism related proteins’ and ‘transcription factors’ terms. Taken together, a total of 121 target genes were selected by combining the RNA-Seq and ChIP-Seq analyses as summarized in Table 1. For the individual OsNAC proteins, we found 72 genes that were up-regulated in the RNA-Seq analysis and whose promoter regions were enriched in the ChIP-Seq analysis. The data suggested that 12, 13, 21 and 26 genes are direct target genes of the individual OsNAC5, 6, 9, and 10 genes, respectively.

**Table 1:** Summary of the integration of RNA-Seq and chromatin immunoprecipitation (ChIP)-Seq data and gene ontology (GO) analysis. ^1^AGRIGO (http://bioinfo.cau.edu.cn/agriGO/). V, vesicle related (GO:0031982, GO:0031988, GO:0016023, GO:0031410); M, membrane related (GO:0043231, GO:0043227). ^2^PANTHER (http://geneontology.org/). TA, transmembrane transport (GO:0055085, GO:0006810, GO:0005215); CM, carbohydrate metabolic process (GO:0005975). ^3^NBRP database (http://shigen.nig.ac.jp/rice/oryzabase/). DT, drought tolerance (Trait Ontology, TO:0000276); GY, grain yield (TO:0000396); RS, root system (Plant Ontology, PO:0025025). Black circle, detected in ChIP-Seq data; Black bold circle, detected in RNA-Seq; Black light circle, not detected in RNA-Seq

Locus	Description	Symbol	RNA-Seq/ChIP-Seq					^1^AgriGO		^4^PANTHER			^8^Oryzabase		
			OsNAC	5	6	9	10								
Transmembrane / Transporter activity															
Os01g0881300	MtN3 and saliva related transmembrane protein family	SWEET1A		**Ο**	Ο										
Os02g0513100	MtN3 protein precursor	SWEET15		Ο	**Ο**			^2^V	^**3**^M	^**5**^T	^**6**^TA				
Os11g0508600	MtN3 protein precursor	SWEET14			**Ο**	Ο	Ο			T	TA				
Os05g0493800	MtN21 nodulin protein-like			Ο		Ο	**Ο**	V	M	T	TA				
Os05g0409500	Usually multiple acids move in and out transporter 9	OsUMAMIT9		**Ο**	Ο	Ο	**Ο**			T	TA				
Os06g0179200	Nodulin-like protein			Ο			**Ο**		M	T	TA				
Os02g0227200	Early nodulin	ENOD93b		Ο		**Ο**			M	T					
Os03g0115000	Cupredoxin domain	ENODL10		Ο			**Ο**		M						
Os06g0681200	Cupredoxin domain	OsENODL18		**Ο**		**Ο**	Ο	V	M				^9^DT		^11^RS
Os03g0146100	Tonoplast intrinsic protein	TIP1;1		**Ο**		**Ο**		V	M						RS
Os02g0658100	Tonoplast membrane integral protein ZmTIP2-1	TIP2;1					**Ο**			T	TA				
Os01g0232000	Major intrinsic protein family	TIP4;3			Ο	Ο	**Ο**	V	M	T	TA				
Os01g0685800	ATP synthase beta chain, mitochondrial precursor				**Ο**	**Ο**	**Ο**		M	T	TA				
Os01g0239200	Phophate translocator	TPT1		**Ο**	Ο		Ο		M	T	TA				
Os02g0496900	Mitochondrial import receptor, TOM9-2 subunit			Ο	**Ο**	Ο	Ο			T	TA				
Os02g0773400	Cyclic nucleotide-binding domain containing protein	OsCNGC12, OsCNGC4			Ο		**Ο**		M	T	TA				
Os02g0693700	ABC transporter superfamily ABCB subgroup member 11	OsABCB11			Ο	**Ο**				T	TA		DT		RS
Os04g0642000	ABC transporter superfamily ABCB subgroup member 16	OsABCB16			Ο		**Ο**			T	TA		DT		
Os05g0424000	amino acid permease 11F	OsAAP11F			**Ο**		**Ο**			T	TA				
Os11g0137000	PIN1-like auxin transport protein	OsPIN1B			**Ο**	Ο	Ο			T	TA				
Os03g0216700	Citrate transporter	OsFRDL1				**Ο**				T	TA				
Os03g0571900	Transparent testa 12 protein	OsPEZ1		**Ο**	**Ο**					T	TA				
Os10g0195000	Multi antimicrobial extrusion protein MatE family	OsSTA240				Ο	**Ο**			T	TA				RS
Os06g0494400	Multi antimicrobial extrusion protein MatE family				**Ο**	Ο				T	TA				
Os02g0510600	Heavy metal transport/detoxification protein domain						**Ο**			T	TA				
Os03g0751600	Heavy metal transport/detoxification protein domain				**Ο**		Ο	V	M	T					
Os08g0403300	Heavy metal transport/detoxification protein domain					**Ο**				T					
Os10g0532300	Heavy metal transport/detoxification protein domain			**Ο**					M	T					
Os01g0849000	non-specific lipid transfer protein d5	OsLTPd5			**Ο**	Ο				T					RS
Os01g0856600	Nsp1-like, C-terminal family			Ο	**Ο**	Ο	**Ο**	V	M	T					
Os07g0198300	Bifunctional inhibitor/plant lipid transfer protein/seed storage domain containing protein.	OsLTPG19		**Ο**		Ο	**Ο**		M	T	TA				
Os12g0104800	Clathrin heavy chain				Ο	Ο	**Ο**	V	M	T					
Os03g0234000	Non-symbiotic hemoglobin 3 (rHb3)	OsNSHB3			Ο	Ο	**Ο**	V	M	T					
Os04g0277400	Membrane bound O-acyl transferase, MBOAT family			Ο	Ο	Ο	**Ο**			T					
Os03g0693700	Oxalate oxidase 1 (Germin)	OXO1		**Ο**				V	M						RS
Os02g0532500	Germin-like protein	OsGLP2-1		**Ο**				V	M						
Os05g0277500	Germin-like protein subfamily 2 member 4 precursor	OsGLP5-2			**Ο**	**Ο**	**Ο**	V	M	T					
Os08g0506400	Serine/threonine protein kinase domain containing protein	OsRLCK257			**Ο**		Ο	V	M				DT		RS
Os05g0371600	BRASSINOSTEROID INSENSITIVE 1-associated receptor kinase 1	OsRLCK184					**Ο**	V	M				DT		
Os04g0576900	Protein kinase-like domain	XIAO			**Ο**	**Ο**	Ο	V	M						
Os03g0416200	BRITTLE CULM1, Cell Wall Architecture1	OsBC1			Ο	Ο	**Ο**	V	M					^10^GY	RS
Os10g0453900	Eggshell protein family	OsGRP7			**Ο**	Ο	**Ο**	V	M						RS
Os01g0668100	Arabinogalactan-like protein	OsFLA7		Ο	Ο		**Ο**	V	M						RS
Os02g0757100	Phi-1 protein	OsPHI-1			**Ο**	Ο	**Ο**	V	M						RS
Os02g0219800	Tetraspanin domain containing protein	OsTET2		Ο	**Ο**	**Ο**	**Ο**	V	M						RS
Os01g0699900	Plasma membrane associated protein-like				**Ο**	Ο	**Ο**	V	M						
Os06g0634500	Leucine-rich repeat transmembrane protein kinase 1			**Ο**	Ο	Ο		V	M						
Os08g0442700	SERK1	COE1		Ο	Ο		**Ο**	V	M						
Os06g0269300	Soluble quinoprotein glucose dehydrogenase domain			Ο		**Ο**	Ο	V	M						
Os12g0568900	Thaumatin, pathogenesis-related family			Ο	Ο		**Ο**	V	M						
Os01g0631500	Beta-1,3-glucanase-like protein				Ο	**Ο**		V	M						
Os04g0579200	Zinc finger, RING-type domain				Ο	Ο	**Ο**	V	M						
Os01g0121800	Glycosyl transferase, family 14 protein			Ο	Ο		**Ο**	V	M						
Os12g0611900	Glycosyl transferase, family 17 protein				**Ο**			V	M						
Carbohydrate Metabolic Process															
Os12g0512100	Sugar transporter family			**Ο**	**Ο**	**Ο**					TA	^**7**^CM			
Os02g0575800	Aldose 1-epimerase family			**Ο**	Ο			V	M			CM			
Os07g0539400	Glycoside hydrolase, family 17 protein, β-1, 3-glucanase 12	Gns12			**Ο**			V	M			CM			
Os04g0376400	Glycoside hydrolase, family 18 protein			Ο	Ο		**Ο**	V	M			CM			
Os02g0790500	Trehalose-6-phosphate synthase	OsTPS5			**Ο**	**Ο**	**Ο**		M			CM			
Os05g0247100	Drought-induced protein 3, Xylanase inhibitor protein 2	DIP3, OsHI-XIP		Ο			**Ο**	V	M			CM	DT		RS
Os07g0646800	Avr9/Cf-9 rapidly elicited protein 231	OsSTA2		**Ο**		**Ο**	Ο		M			CM			RS
Os01g0675500	Glycoprotein-specific UDP-glucuronyltransferase-like protein	OsGT43F				Ο	**Ο**					CM			
Os08g0237800	Xyloglucan endotransglycosylase	OsXTH8		Ο	Ο		**Ο**	V	M			CM			RS
Os06g0356800	Xylanase inhibitor protein I precursor			**Ο**				V	M			CM			
Os02g0764200	Xyloglucan fucosyltransferase-like					**Ο**			M			CM			
Os06g0212300	Xyloglucan fucosyltransferase-like			Ο	Ο		**Ο**		M			CM			
Os01g0944700	Beta-1,3-glucanase precursor	OsGLN2			**Ο**		Ο	V	M			CM			
Os07g0480800	Xyloglucan endotransglucosydase / hydrolase 21	OsXTH21		**Ο**	**Ο**	Ο		V	M			CM			
Os04g0556400	UDP-glycosyltransferase	UGT93B9		**Ο**	Ο	Ο	Ο					CM			
Os04g0486950	Malate synthase						**Ο**					CM			
Os07g0490100	UDP-glucuronosyl / UDP-glucosyltransferase family	UGT709A4		Ο	**Ο**		Ο	V	M			CM			
Os01g0276800	Mannose-6-phosphate receptor, binding domain containing protein						**Ο**	V	M			CM			
Os01g0329300	Pectin lyase fold domain containing protein				Ο	**Ο**			M			CM			
Os03g0421800	X8 domain containing protein			Ο			**Ο**	V	M						
Auxin / Hormon related															
Os08g0109400	AUX/IAA protein family	OsIAA25			**Ο**	**Ο**			M						
Os02g0769100	Auxin responsive SAUR protein family	OsSAUR12		Ο	Ο		**Ο**		M						
Os06g0714300	Auxin responsive SAUR protein family	OsSAUR29			**Ο**	Ο	Ο		M						
Os02g0167600	Auxin responsive SAUR protein family	OsSAUR6		Ο			**Ο**								
Os05g0381400	ABA induced plasma membrane protein PM 19	OsPM19L1		**Ο**	Ο	**Ο**	Ο	V	M				DT		RS
Os01g0859300	ABA response element binding factor	OsABI5		**Ο**	Ο	**Ο**			M						RS
Os03g0760800	GAST1 protein precursor	OsGASR1		**Ο**			Ο	V	M						RS
Os05g0432200	Gibberellin-regulated protein 2 precursor			Ο	Ο	**Ο**	Ο	V	M						
Transcription factors															
Os07g0227600	Pathogenesis-related transcriptional factor and ERF domain	OsERF57				**Ο**			M						
Os01g0657400	Ethylene-responsive transcription factor 5	OsERF54			**Ο**				M						
Os03g0263000	TINY-like protein	OsERF9					**Ο**		M						
Os02g0521100	Non-protein coding transcript, unclassifiable transcript	OsERF107				**Ο**									
Os06g0222400	Conserved hypothetical protein	OsERF120					**Ο**		M						
Os04g0429050	Ethylene response factor 100	OsERF100		**Ο**											
Os05g0541400	Helix-loop-helix DNA-binding domain containing protein	OsbHLH119			**Ο**										
Os05g0139100	Helix-loop-helix DNA-binding domain containing protein	OsbHLH106			Ο	**Ο**			M						
Os03g0782500	Phytochrome-interacting factor-like bHLH protein, Stress-responsive transcription factor	OsbHLH152		**Ο**					M						
Os10g0376900	Helix-loop-helix DNA-binding domain containing protein	OsbHLH045			Ο	**Ο**	Ο		M						
Os06g0613500	Helix-loop-helix DNA-binding domain containing protein	OsbHLH095				Ο	**Ο**		M						
Os04g0641700	INCREASED LEAF INCLINATION 1	OsbHLH154		Ο	Ο	Ο	**Ο**							GY	RS
Os01g0854500	Homeobox domain containing protein	OsWOX5			Ο		**Ο**		M				DT		RS
Os01g0182700	WRKY GENE 102	WRKY102			**Ο**	Ο			M				DT		
Unclassified															
Os07g0604433	Glycosyltransferase family-37			Ο	Ο		**Ο**								
Os05g0370300	C2 calcium/lipid-binding region, CaLB domain			**Ο**			Ο		M						
Os03g0161400	IQ calmodulin-binding region domain			Ο	Ο	Ο	**Ο**		M						
Os01g0833800	IQ calmodulin-binding region domain				Ο		**Ο**								
Os07g0619400	Calcium-binding EF-hand domain			Ο	**Ο**	Ο	Ο								
Os07g0622700	Alpha/beta hydrolase fold-1 domain containing protein				Ο	**Ο**	Ο		M						
Os12g0119800	Harpin-induced 1 domain					**Ο**	Ο		M						
Os09g0419200	NAD-dependent epimerase/dehydratase family			Ο	Ο	**Ο**			M						
Os03g0136900	Aconitate hydratase, cytoplasmic			**Ο**	Ο	Ο			M						
Os01g0330100	GDSL ESTERASE/LIPASE PROTEIN 15	OsGELP15		Ο	Ο		**Ο**		M						
Os09g0527500	MEG5				Ο	Ο	**Ο**		M						
Os07g0601000	NADPH HC toxin reductase			Ο		Ο	**Ο**		M						
Os01g0618700	Lipase, class 3 family			Ο		Ο	**Ο**		M						
Os01g0745400	Sec34-like protein family	Sec34-like			Ο	**Ο**	**Ο**								
Os08g0545200	Alcohol dehydrogenase superfamily	OsDH1		Ο	Ο		**Ο**								
Os06g0727200	Catalase isozyme B	OsCAT1B		Ο	Ο		**Ο**								
Os03g0110500	KIP1-like domain			**Ο**	**Ο**	**Ο**	Ο								
Os06g0704500	Leucine-rich repeat, plant specific				Ο	**Ο**	Ο								
Os01g0613500	Peptidase C1A, papain family			Ο	Ο	Ο	**Ο**								
Os12g0613600	Remorin, C-terminal region domain containing protein	OsREM6.3		Ο		**Ο**	Ο								
Os01g0695300	Farnesyl pyrophosphate synthetase	FPS			Ο	Ο	**Ο**								
Os01g0634500	Laccase-2				Ο	Ο	**Ο**								
Os03g0392050	Oligosaccaryltransferase domain containing protein			Ο	Ο	Ο	**Ο**								
Os07g0605350	Protein EGG APPARATUS-1				Ο	**Ο**	Ο								
Os12g0127650	Ulp1 protease family, C-terminal catalytic domain			Ο	Ο	Ο	**Ο**								

**Fig. 7:**
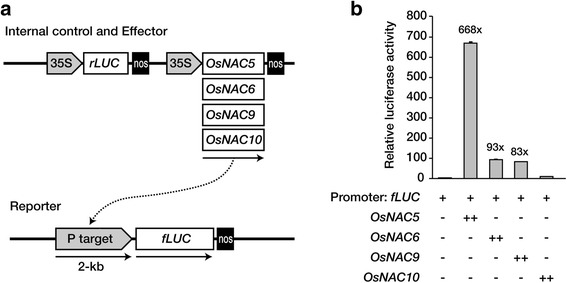
OsNAC-mediated transactivation of abscisic acid (ABA) response element binding factor (Os01g0859300) gene. **a** Construction of vectors for rice protoplast transactivation assays of the Os01g0859300 gene with OsNAC5, 6, 9, or 10. **b** Fold-changes are normalized relative to protoplasts co-transfected only with the reporter and internal control constructs. Firefly luciferase (fLuc) activities were normalized relative to those of renilla luciferase (rLuc). The error bars designate SD (standard deviation) of the mean (n = 3)

To assess whether the OsNAC proteins can bind to the promoter region and activate the transcription of the target gene, we performed a transient expression assay in rice protoplasts using a dual-luciferase reporter system. We have chosen one target gene, *OsABI5* (Os01g0859300) that was found to be regulated by OsNAC5, 6 and 9, but not by OsNAC10. A reporter plasmid with the 2-kb promoter region of *ABI5* (Os01g0859300) fused to a firefly luciferase gene was constructed (*pABI5:fLuc*, Fig. 7a). Effector plasmids with *OsNAC5*, *6*, *9*, and *10*, and a *CaMV35S:rLuc* (renilla luciferase) expression cassette as an internal control, were constructed (Fig. 7a). These plasmids were transiently co-expressed in rice protoplasts in the combinations indicated in Fig. 7b. Expression of the reporter gene was strongly activated by OsNAC5, 6, and 9, but not by OsNAC10 (Fig. 7b), validating the results of the RNA-Seq and ChIP-Seq analyses.

## Discussion

Resolving TF networks, including the identification of direct target genes, is a common goal in functional genomic and molecular biology studies. Direct target genes have previously been identified using in vitro methods, such as the electrophoretic mobility shift assay (EMSA) and yeast one-hybrid analysis. However, the ChIP method has emerged as a powerful tool to detect interactions between DNA-binding proteins and genomic DNA. Our group has characterized the drought-inducible TFs, *OsNAC5, 6, 9* and *10* [25, 29–31], overexpression of these genes specifically in transgenic rice roots (*RCc3:OsNAC5, 6, 9* or *10*) caused enlarged root diameter and drought tolerance. Putative target genes involved in root growth, development and abiotic stress response were identified in the roots of these plants by microarray [25, 30, 31] or RNA-Seq [29] analyses. However, it is difficult to identify a regulatory network or a specific pathway using such data due to a lack of information about binding interactions between TFs and target genes [35, 36].

In this study, we performed RNA-Seq and ChIP-Seq analysis of roots of *RCc3:6MYC-OsNAC5*, *6*, *9* and *10* plants and identified direct OsNAC target genes by cross-referencing transcriptome (RNA-Seq) information and binding loci (ChIP-Seq) information. The RNA-Seq experiments led us to identify 45 up- and 213 down-regulated genes in all four *RCc3:6MYC-OsNAC* transgenic roots (Additional file 3: Table S2). These included genes involved in the ‘cellular components’, ‘vesicles’, ‘cytoplasmic membrane-bounded vesicle’, and ‘cytoplasmic vesicle’ GO terms (Additional file 4: Table S3). We previously demonstrated that the *RCc3:OsNAC* transgenic roots had increased root diameter due to an enlarged xylem and augmented cortical cells. These changes in root phenotype appeared to be correlated with the up-regulation of genes involved in signal transduction (abscisic acid and Ca^2+^), barrier formation (lignin and suberin biosynthesis), and cell development and morphogenesis [25, 30, 31]. However, RNA-Seq data alone provide no information about whether the target genes are regulated directly by the TF binding to *cis*-elements of the target gene promoter, or indirectly through regulation of other TFs or signaling molecules. To determine the direct targets of the four OsNAC TFs, a genome-wide analysis of the interactions of OsNAC5, 6, 9, and 10 with the promoters of regulated genes was performed. Cross-referencing of the ChIP-Seq and RNA-Seq data sets from the four *RCc3:OsNAC* root samples allowed us to identify 391 direct OsNAC5, 6, 9, and 10 target genes (Additional file 9: Table S7). Our ChIP-Seq data revealed that 158, 202, 198, and 239 loci were bound by OsNAC5, 6, 9, and 10, respectively, and that 65, 91, 115, 186 genes, respectively, were up-regulated (Fig. 6). Of the up-regulated genes in the RNA-Seq experiments, 8.7, 6.5, 7.4, and 4.0% were direct targets in the *RCc3:6MYC-OsNAC5*, *6*, *9* and *10* roots, respectively. The direct target genes could be divided into 5 subgroups according to GO analysis: ‘transmembrane/transporter activity’, ‘vesicle related’, ‘hormone related’, ‘carbohydrate metabolic process’, and ‘TFs’ (Table 1). Of the genes encoding transmembrane proteins that were directly regulated by the OsNAC TFs, PIPs (plasma membrane intrinsic proteins) and TIPs (tonoplast intrinsic proteins) are known to be more abundantly expressed in roots than in leaves, and their expression responds to drought stress, contributing to the drought tolerance of the *RCc3:6MYC-OsNAC5*, *6*, *9* and *10* plants. Moreover, their localization is associated with the presence of root hydrophobic barriers [37]. This idea is supported by the observation that transgenic *A. thaliana* lines overexpressing the *PIP1* gene from *Panax ginseng* [38], TIP1;2 from the highly drought-resistant *Thellungiella salsuginea* [39] and *TIP2* from *Solanum lycopersicum* [40] exhibited higher survival rates, reduced water loss, and tolerance of water deprivation under drought conditions. The TIPs might help promote increases in turgor pressure and cell elongation, and the mediation of water transport across cell membranes, including the tonoplast, in response to drought [41, 42]. Together, these proteins are involved in controlling water movement during drought stress. NODULIN genes, which we found to be directly regulated by the OsNAC TFs, are from the MtN3/SALIVA/SWEET family (Os01g0881300, Os11g0508600, Os02g0513100), MtN21/EamA-like TRANSPORTER/UMAMIT family (Os05g0409500), and EARLY NODULIN-LIKE (ENODL) family (Os03g0115000 and Os06g0681200). Furthermore, in *A. thaliana*, the sugar transporter *SAG29/SWEET15* (At5g13170) is a direct downstream target of an AtNAC TF, ANAC092/AtNAC2/ORE1 [43]. We also identified an ENODL gene (Os06g0681200), which is associated with root architecture by trait ontology in the NBRP database (http://shigen.nig.ac.jp/rice/oryzabase/), as being involved in drought tolerance (Table 1). Among the direct targets were also four genes (Os03g0216700, Os03g0571900, Os10g0195000 and Os06g0494400) encoding a multi antimicrobial extrusion proteins and four genes (Os02g0510600, Os03g0751600, Os08g0403300, and Os10g0532300) encoding heavy metal transport/detoxification proteins (Table 1). It has previously reported that the metal stress signaling pathway is associated with controlling drought stress signaling [44], and that the genes involved encode transmembrane/transporters that control physiological processes essential for optimal plant growth, as well as for abiotic stress tolerance.

Other subgroups of direct OsNAC targets that have previously been shown to be involved in drought tolerance include the drought-inducible germin-like proteins, of which there are ten in rice [45]. Germin-like proteins are divided into two groups based on oxalate oxidase and superoxide dismutase activities, which catalyzes the oxidative breakdown of oxalate to CO_2_ and H_2_O_2_. They are important in cell wall fortification, mediating auxin responsive physiological processes [41, 46], and germin proteins have also been suggested to contribute to the preparation of cell walls [27] by producing H_2_O_2_ required by peroxidase to catalyze the crosslinking of several cell wall components [28]. Thus, germin and germin-like proteins play a role in strengthening plant cell walls to resist abiotic stress [34, 39, 40]. Zhong et al. (2010) showed that SND1 and VND7, two *A. thaliana* NAC-domain TFs, directly regulate the expression of not only downstream TFs, but also genes involved in the secondary wall biosynthesis and polysaccharide biosynthesis [47]. Similarly, we found that the OsNAC TFs targeted in this study directly regulate genes involved in cell wall polysaccharide biosynthesis (Table 1). Two types of TFs, AP2/EREBP and bHLH, were among the direct targets (Table 1). Six AP2/EREBP- and six bHLH-domain genes were directly regulated by one of the four OsNAC TFs, such as *OsERF1* (Os04g0429050) in *RCc3:OsNAC5*, *OsERF54* (Os01g0657400) in *RCc3:OsNAC6*, *OsERF57* (Os07g0227600) and *OsERF107* (Os02g0521100) in *RCc3:OsNAC9*, and *OsERF120* (Os06g0222400) and *OsERF9* (Os03g0263000) in *RCc3:OsNAC10*. A similar number of the bHLH TF genes were regulated in the transgenic rice roots (Table 1). Thus, drought inducible OsNAC TFs bind to their target gene promoters, up-regulating different classes of TFs including the AP2/EREBP- and bHLH-domain genes, indicating a role in the crosstalk inherent in abiotic stress responses.

Previous studies have predicted the functional redundancy of OsNAC5, 6, 9, and 10 by observing similar root phenotypes such as an enlarged root and drought tolerance in the OsNAC overexpressors [25, 26–28]. In the current manuscript, we suggest the functional redundancy of OsNACs (Fig. 6) by the target gene expression profiles in the OsNAC overexpressors. We present a strategy to identify direct OsNAC target genes involving ChIP-Seq and RNA-Seq analyses of *RCc3:OsNAC5, 6, 9,* and *10* expressing roots, which are drought tolerant. These results were validated using transient expression assays in rice protoplasts using a dual-luciferase reporter system (Fig. 7). Many of the direct target genes have previously been shown to be involved in drought tolerance, highlighting the important roles of the four OsNAC TFs in the associated gene networks.

## Conclusions

Abiotic stress triggers a wide range of plant responses, from the alteration of gene expression and cellular metabolism to change in plant growth and development. Thus, understanding the complex mechanism of drought tolerance is crucial for crop production. Uncovering the molecular networks of the TFs that are involved in drought tolerance provides information about the individual processes of how the TFs interact in a drought signaling pathway. We obtained 391 direct targets of OsNACs by comparing genes from the results of RNA-Seq with those of ChIP-Seq. Among the genes selected from RNA-Seq results, the ratio of becoming a direct target of OsNACs ranged from 4.0% to 8.7% depending on *RCc3:OsNAC*s plants. Interestingly, OsNACs target several cellular components, including transmembrane/transporter, vesicle, auxin/hormone, carbohydrate metabolic processes and transcription factors. Many of the target genes that are up-regulated by OsNACs act as the cellular components which would alter the root architectures of *RCc3:OsNAC*s for drought tolerance. This would be a valuable resource for the functional dissection of the molecular mechanisms in plant drought response.

## Additional files

Additional file 1: Table S8. List of primers used in this study. (XLSX 12 kb)

Additional file 2: Table S1. Information about RNA-Seq data obtained by Illumina Hi-Seq 2500 sequencing. ^1^Raw seq. Reads no.: Total number of reads used in sequence analysis. ^2^Raw seq. Reads length: Total length of reads used in sequence analysis. ^3^GC (%): Percentage of GC found in sequence reads. ^4^Q20 (%): Percentage of bases with quality score of 20 or above. A base with a quality score of 20 has a probability of being an incorrect call of 1 in 100. (XLSX 14 kb)

Additional file 3: Table S2. List of up- and down-regulated differentially expressed genes (DEGs) (|fold change| ≥ 1.5, and *p* value <0.05,) in *RCc3:OsNAC5, 6, 9,* and *10* rice plants. (XLSX 54 kb)

Additional file 4: Table S3. Gene ontology analysis of genes that are up-regulated in *RCc3: OsNAC5, 6, 9,* and *10* transgenic roots. Genes were associated with gene ontology (GO) terms, including Biological Process, Molecular Function, and Cellular components. FDR, False Discovery Rate, *p*-value <0.05 using AgriGO and REVIGO. Main enriched processes are presented for each of RCc3:OsNAC5, 6, 9, and 10. (XLSX 15 kb)

Additional file 5: Table S4. List of genes associated with gene ontologies, including Biological Process, Molecular Function, and Cellular components. (XLSX 152 kb)

Additional file 6: Table S5. ChIP-Seq data obtained by Illumina Hi-Seq 2500 sequencing. (XLSX 16 kb)

Additional file 7: Table S6. Direct target genes of OsNAC5, 6, 9, and 10 identified by merging of chromatin immunoprecipitation (ChIP)-Seq and RNA-Seq data sets. Each color showed that Log2 ratio ≥ 1.0 in ChIP, and f.c. (fold change) ≥ 1.5, p-value <0.05 in RNA-seq, and red, sky blue, light green, and purple mean OsNAC5, OsNAC6, OsNAC9, and OsNAC10, respectively. 975 sheet means a total of 975 genomic loci, including 460 promoters, 11 5’ UTR, 211 exons, 116 introns, 148 TTs, and 28 3’ UTRs, and 460 sheet means 460 promoters that were enriched in at least one of the OsNACs. Distance to TSS, the distance between the nearest TSS and the center of the peak/region. (XLSX 1228 kb)

Additional file 8: Figure S1. Singular enrichment analysis was performed using AgriGO (http://bioinfo.cau.edu.cn/agriGO/) to identify enriched gene ontologies up-regulated in OsNAC transgenic rice plants. a. OsNAC5, b. OsNAC6, c. OsNAC9, d. OsNAC10. (TIFF 4245 kb)

Additional file 9: Table S7. Integration of RNA-Seq and chromatin immunoprecipitation (ChIP)-Seq data sets. (XLSX 36 kb)

## Abbreviations

ChIP-Seq: chromatin immunoprecipitation sequencing
qRT-PCR: quantitative reverse transcribed polymerase chain reaction
RNA-Seq: RNA sequencing
