# Supplementary figures and images for: Genome-wide analyses of direct target genes of four rice NAC-domain transcription factors involved in drought tolerance
Source: BMC Genomics. 2018 Jan 12;19:40. doi: 10.1186/s12864-017-4367-1 (PMC5767043; doi:10.1186/s12864-017-4367-1)

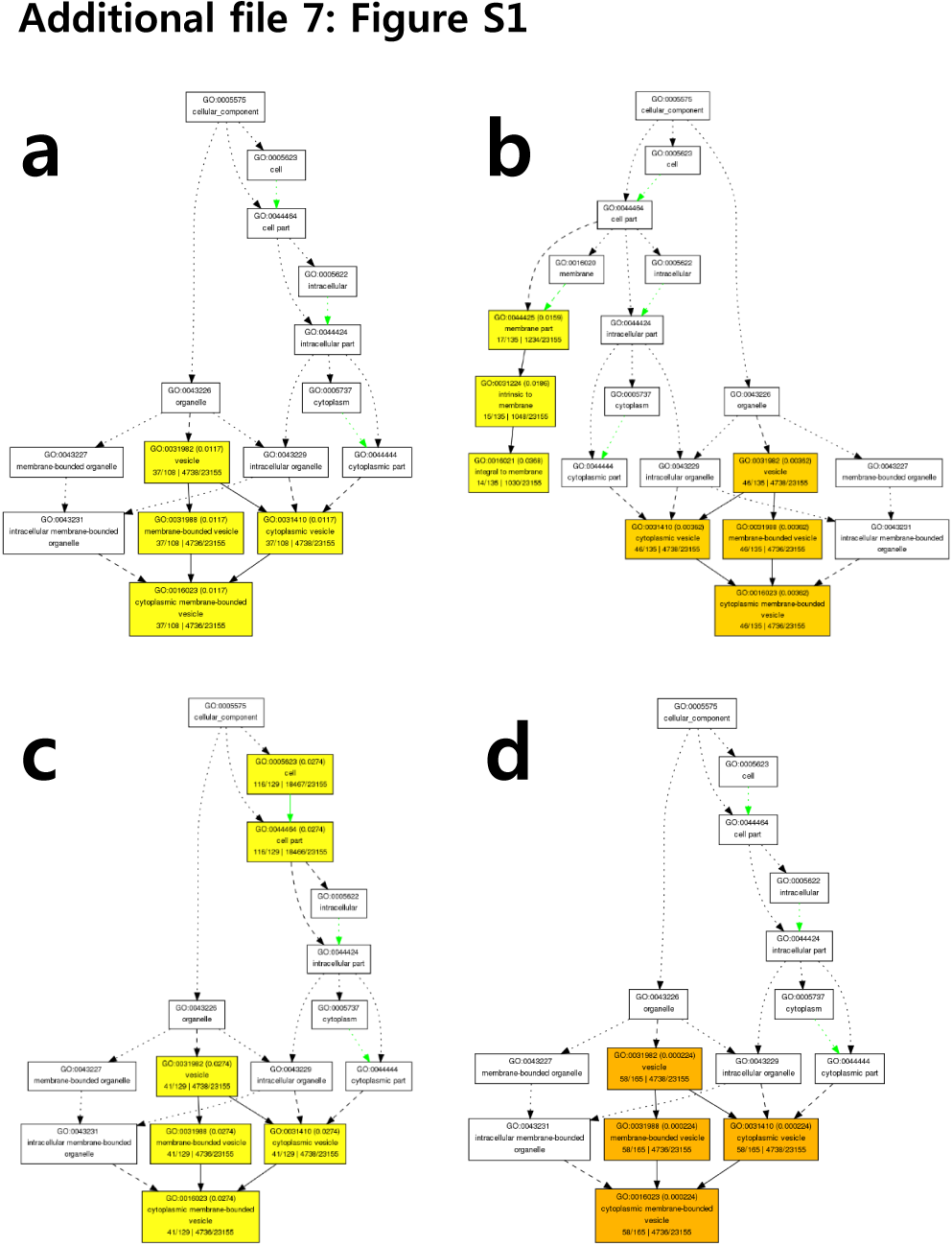

Supplement: Supplementary file 8 — Singular enrichment analysis was performed using AgriGO (http://bioinfo.cau.edu.cn/agriGO/) to identify enriched gene ontologies up-regulated in OsNAC transgenic rice plants. a. OsNAC5, b. OsNAC6, c. OsNAC9, d. OsNAC10. (TIFF 4245 kb) [file 12864_2017_4367_MOESM8_ESM.tif]
